# Azidation in the Difunctionalization of Olefins

**DOI:** 10.3390/molecules21030352

**Published:** 2016-03-16

**Authors:** Kai Wu, Yujie Liang, Ning Jiao

**Affiliations:** State Key Laboratory of Natural and Biomimetic Drugs, School of Pharmaceutical Sciences, Peking University, Xue Yuan Rd. 38, Beijing 100191, China; kekoms@163.com (K.W.); liangyj_pku@163.com (Y.L.)

**Keywords:** azides, difunctionalization, olefins

## Abstract

Organic azides are key motifs in compounds of relevance to chemical biology, medicinal chemistry and materials science. In addition, they also serve as useful building blocks due to their remarkable reactivity. Therefore, the development of efficient protocols to synthesize these compounds is of great significance. This paper reviews the major applications and development of azidation in difunctionalization of olefins using azide reagents.

## 1. Introduction

The first organic azide, phenyl azide, discovered by Griess in 1864 opened the door for the use of organic azides in synthesis [1]. Since then, numerous elegant approaches have been developed, such as the *aza*-Wittig reaction [2], Sundberg rearrangement, Curtius rearrangement [3,4], Schmidt rearrangement [5], Hemetsberger rearrangement [6] and click chemistry [7,8,9,10,11,12]. Organic azides are important building blocks and intermediates, not only due to the fact they are potential precursors of N-containing structural motifs [13,14,15,16,17,18,19], but also for their remarkable biological activity in pharmaceutical chemistry [20]. Moreover, organic azides have also received great attention in other fields, such as supramolecular chemistry [21,22,23,24], medicinal chemistry [25], biotechnology and materials science [26].

Olefins are readily available starting materials in organic synthesis. Recently, the difunctionalization of olefins [27,28,29,30,31,32,33,34,35,36,37,38,39,40,41,42] which could introduce two chemical bonds into substrates simultaneously, has attracted considerable attention. Organic azide-driven difunctionalization of olefins has been a hot topic in this area in recent years. Herein, we summarize the recent developments in the use of azidation reactions in the difunctionalization of olefins.

## 2. The Application of Organic Azides in Difunctionalization of Olefins

### 2.1. Construction of C-N_3_ Bond and C-C Bond

In the past decade, the hydroazidation of olefins has been significantly developed [43,44,45,46,47,48], although this kind of addition reaction has not been generally classified as a traditional difunctionalization of olefins. Therefore, we start here from the construction of C-N_3_ bonds and C-C bonds.

**Scheme 1:**
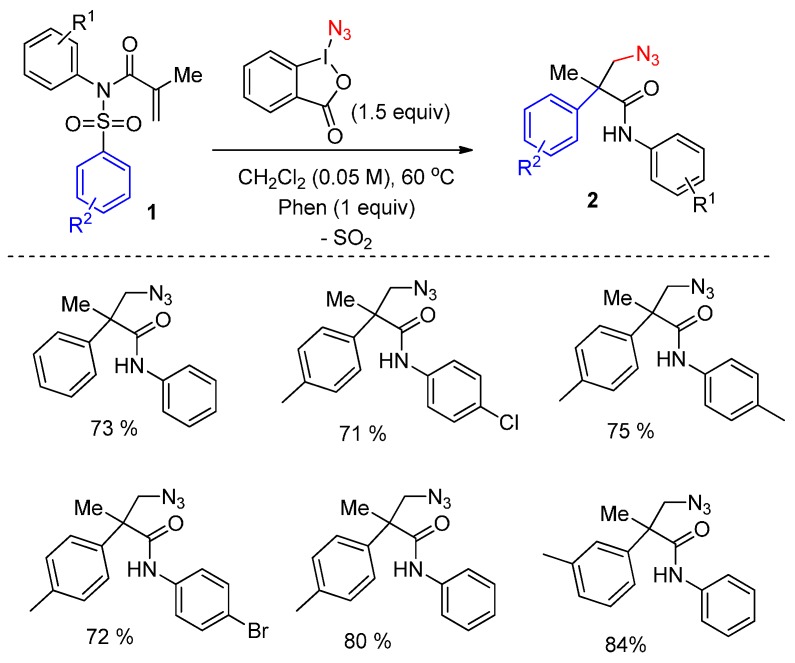
Arylheterofunctionalization of activated alkenes.

An interesting radical-mediated arylazidation of activated alkenes **1** was reported by Nevado and co-workers (Scheme 1) [49]. This process involves radical addition, Csp^2^-Csp^3^ and C-N bond formation, 1,4-aryl migration, and further desulfonylation, producing in good yields the corresponding products **2** bearing a quaternary stereocenter when the substituent on the nitrogen atom is an aryl group.

**Scheme 2:**
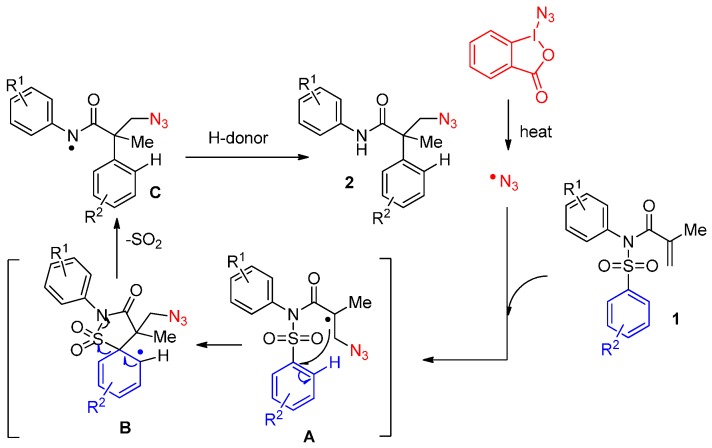
The proposed mechanism of the arylheterofunctionalization of alkenes.

The proposed mechanism is illustrated in Scheme 2. In the first step, the generated azido radical interacts with the activated alkene to give a new C(sp^3^)-N bond and an alkyl radical intermediate **A**. A 5-ipso cyclization then takes place on the aromatic ring generating aryl radical **B**, which leads to amidyl radical **C** upon rearomatization with concomitant desulfonylation. The subsequent H-abstraction step produces the desired product **2** (Scheme 2).

**Scheme 3:**
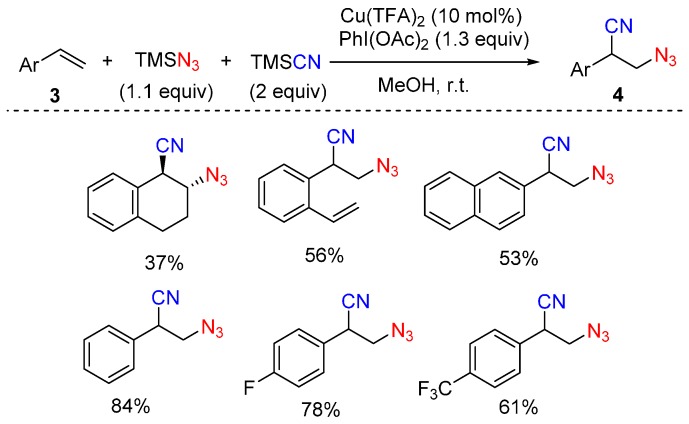
Azidocyanation of aryl alkenes.

Wang and co-workers reported a copper-catalyzed intermolecular Markovnikov-type azidocyanation reaction of alkenes **3** to construct C-N_3_ and C-CN bonds simultaneously (Scheme 3) [50], which gives a series of 3-azido-2-arylpropanenitriles **4** in moderate to good yields. These compounds may serve as potential precursors of the corresponding 3-amino-2-arylpropanoic acids. This reaction employs PhI(OAc)_2_ as oxidant, TMSN_3_ as N_3_ source, TMSCN as CN source and it proceeds at room temperature. A mechanistic study revealed that the addition of 2,6-di-*tert*-butyl-4-methylphenol (BHT) significantly suppressed the reaction, suggesting that a radical process may be involved in this reaction.

**Scheme 4:**
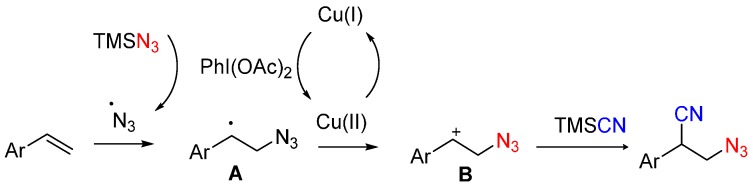
Plausible mechanism of the reaction.

On the basis of the above results, a plausible mechanism is proposed. First, TMSN_3_ could react with PhI(OAc)_2_ to generate the N_3_ radical under the standard reaction conditions. Then the radical addition to alkene gives intermediate **A**. In the presence of copper(II) catalyst, **A** could be oxidized to form the intermediate **B** with a carbocation center, which is trapped by TMSCN to give the final product (Scheme 4).

**Scheme 5:**
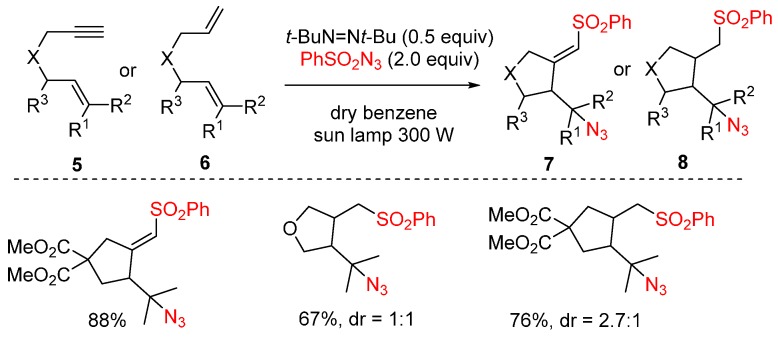
Azidoarylation of alkenes.

A radical mediated azidosulfonylation of various 1-en-6-ynes **5** or 1,6-dienes **6** that are able to undergo a rapid radical rearrangement was reported by Renaud and co-workers (Scheme 5) [51]. This reaction is initiated by di-*tert*-butyldiazene upon irradiation with a 300 W sun lamp. Under the standard reaction conditions, the reaction is generally completed within 2–4 h.

**Scheme 6:**
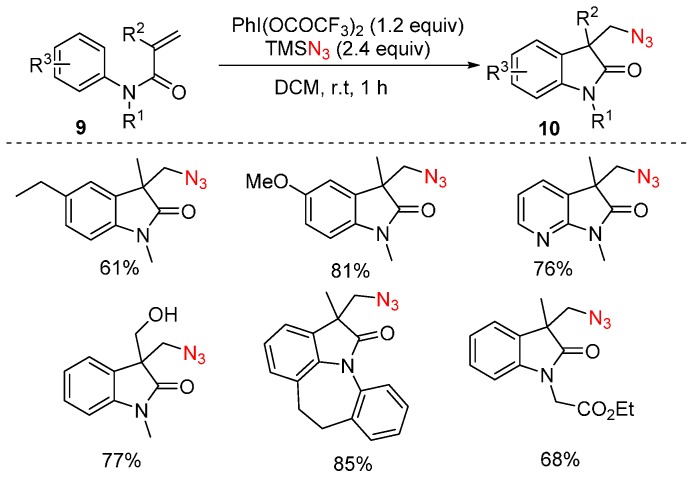
Azidoarylation of alkenes.

2-Oxindoles not only have unique biological activity, but also are important building blocks in drug design and organic synthesis [52,53]. In order to synthesize these useful intermediates, a rapid approach to oxindoles through C-N_3_ and C-C bond construction was reported by Antonchick and co-workers [54]. This chemistry proceeds under mild and metal-free conditions, giving biologically interesting 2-oxindoles **10** in good yields (Scheme 6).

**Scheme 7:**
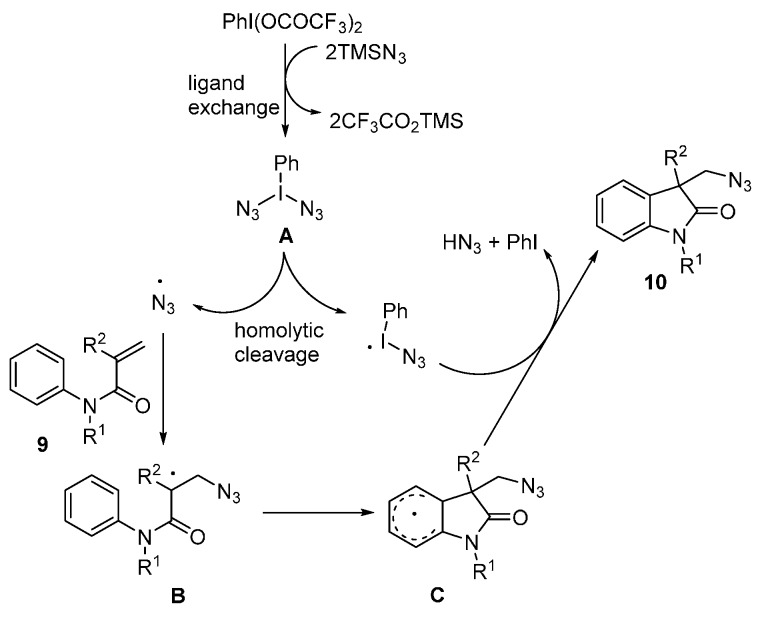
Proposed mechanism for the azidoarylation of alkenes.

**Scheme 8:**
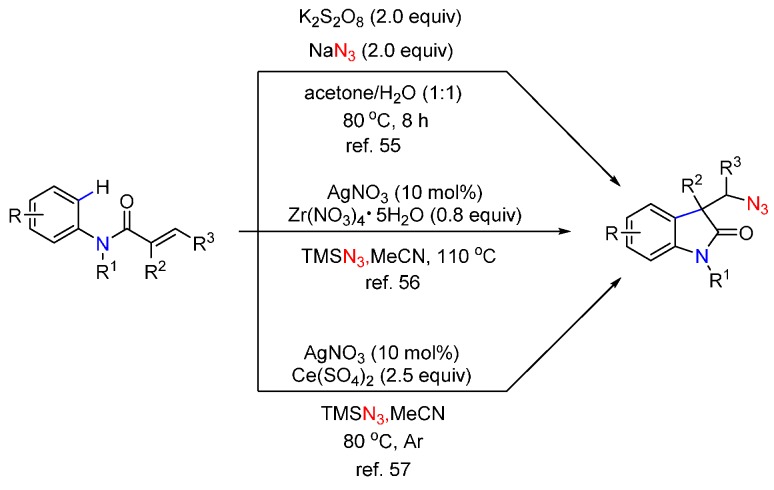
Azidoarylation of alkenes.

A plausible mechanism for this transformation is illustrated in Scheme 7. This reaction begins with a double ligand exchange between PhI(OCOCF_3_)_2_ and TMSN_3_ to provide intermediate **A**, which undergoes thermal homolytic cleavage to generate an azido radical. This azido radical attacks alkene **9** to give intermediate **B**, which is trapped by arene to give intermediate **C**. Rearomatization of **C** provides product **10**. Similar approaches using different catalysts and oxidants were also reported by the groups of Zhang [55], Yang [56], and Jiao [57] (Scheme 8).

**Scheme 9:**
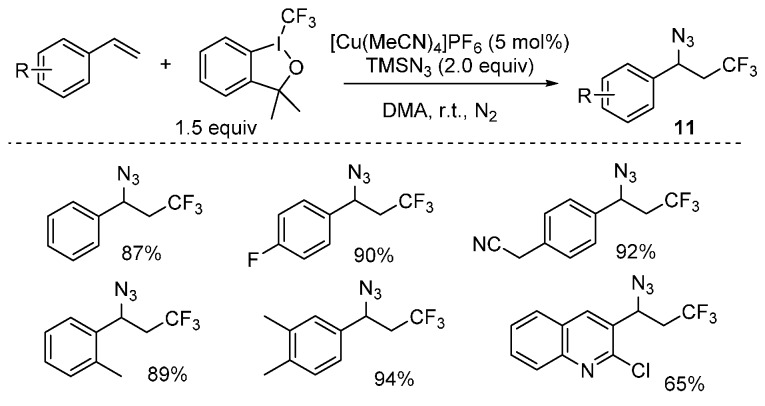
Azidotrifluoromethylation of alkenes.

The introduction of a trifluoromethyl group into chemical compounds is an important way to modify their activities and biocompatibilities [58]. In light of their importance in medicinal chemistry, a novel copper-catalyzed intermolecular azidotrifluoromethylation of alkenes has been developed by Liu and co-workers [59]. By using this method, various CF_3_-containing organoazides **11** were obtained in good yields under mild reaction conditions (Scheme 9). Besides, the resulting products can be readily transformed into other valuable CF_3_-containing amine compounds.

**Scheme 10:**
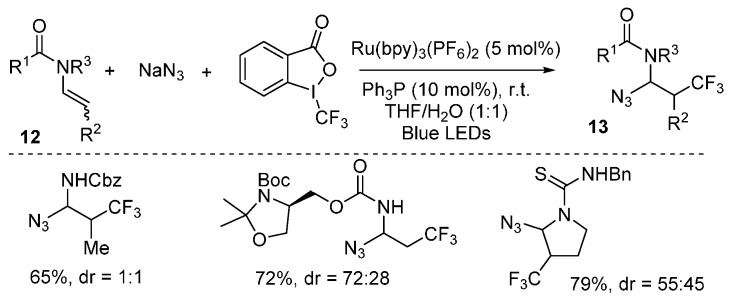
Azidotrifluoromethylation of enecarbamates.

A photoredox-catalyzed azidotrifluoromethylation of enecarbamates **12** was reported by Magnier, Masson and coworkers [60]. This reaction used Tongi’s reagent as CF_3_ source, and was proposed to follow a radical/cationic pathway. Under the optimized conditions, a wide range of substrates can be readily difunctionalized (Scheme 10).

**Scheme 11:**
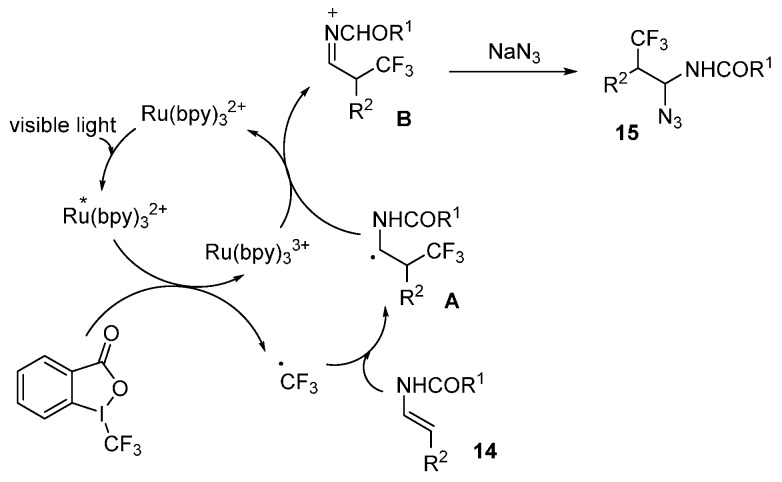
Proposed mechanism.

A possible mechanism is shown in Scheme 11. Visible light excites Ru(bpy)_3_^2+^ into *Ru(bpy)_3_^2+^, which is a strong reductant species that performs a SET to generate trifluoromethyl radical from Togni’s reagent. Then, addition of trifluoromethyl radical to enecarbamate **14** leads to an α-amido radical intermediate **A**, which can be oxidized into N-acyliminium cation intermediate **B** by SET process from Ru(bpy)_3_^3+^. Final nucleophilic trapping by NaN_3_ forms the products **15**.

**Scheme 12:**
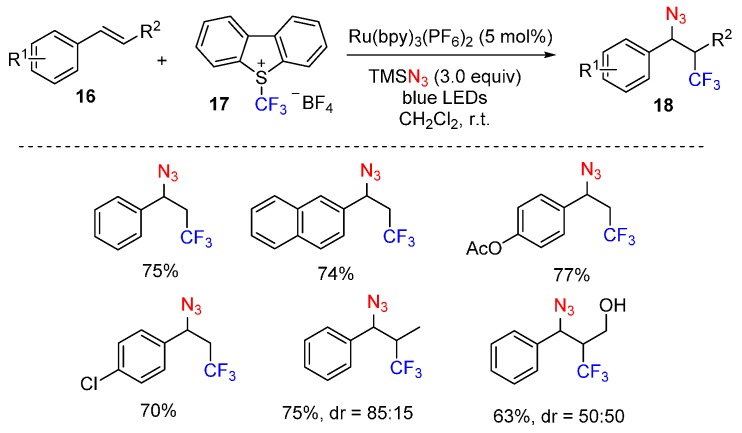
Azidotrifluoromethylation of alkenes.

Inspired by their above work, this group also developed a photoredox-catalyzed azidotrifluoromethylation of alkenes **16**. Under the optimized conditions, using Umemoto’s reagent as the CF_3_ source, a wide range of alkenes can be readily difunctionalized (Scheme 12) [61].

**Scheme 13:**
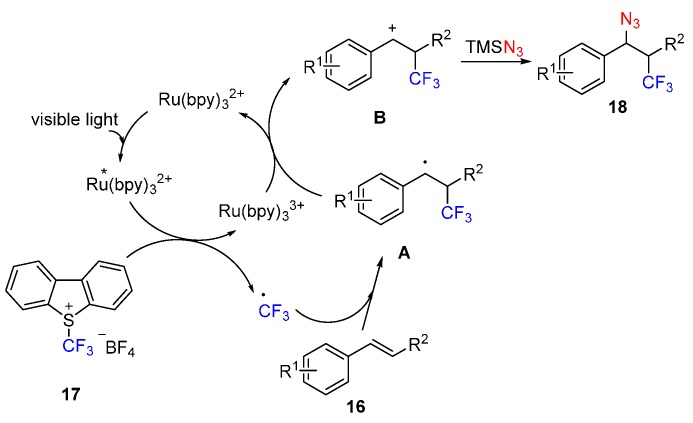
Plausible reaction mechanism.

A similar mechanism is proposed based on their above work (Scheme 13). Firstly, visible light excites the Ru(bpy)_3_^2+^ into *Ru(bpy)_3_^2+^, which performs a SET to generate trifluoromethyl radical from Umemoto’s reagent **17**. Then addition of trifluoromethyl radical to alkenes **16** produces carbon radical intermediate **A**, which can be oxidized into β-trifluoromethylated carbocation intermediate **B**. Finally, carbocation **B** is trapped by TMSN_3_ to form the product **18**.

**Scheme 14:**
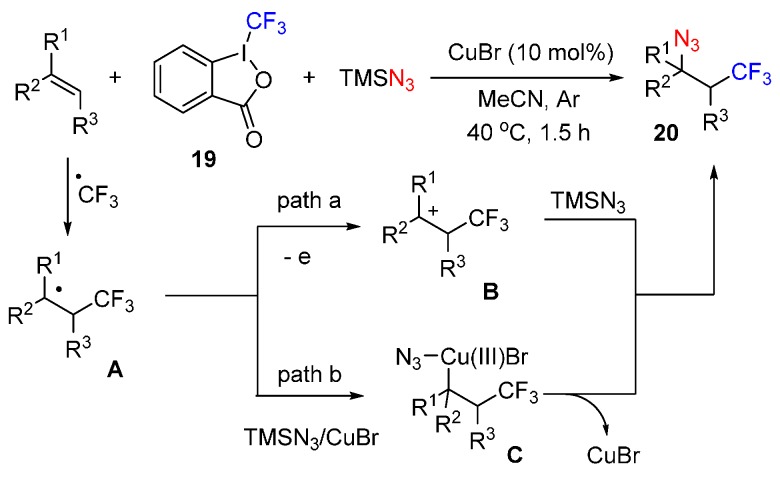
Azidotrifluoromethylation of alkenes.

Due to the importance of these compounds, a copper-catalyzed three-component azidotrifluoromethylation of alkenes was designed by Yang (Scheme 14) [62]. This reaction proceeded under mild conditions and gave the corresponding product **20** in good yields. A possible mechanism was also proposed. Togni’s reagent **19** is activated by CuBr, leading to the radical species **A** by reaction with the alkene. Then, intermediate **A** is oxidized to intermediate **B**, which is further trapped by TMSN_3_ to lead to the product (Path a). Alternatively, intermediate **A** reacts with TMSN_3_ and CuBr to afford complex **C**, further reductive elimination of complex **C** affords the product **20**.

### 2.2. Construction of C-N_3_ Bond and C-O Bond

**Scheme 15:**
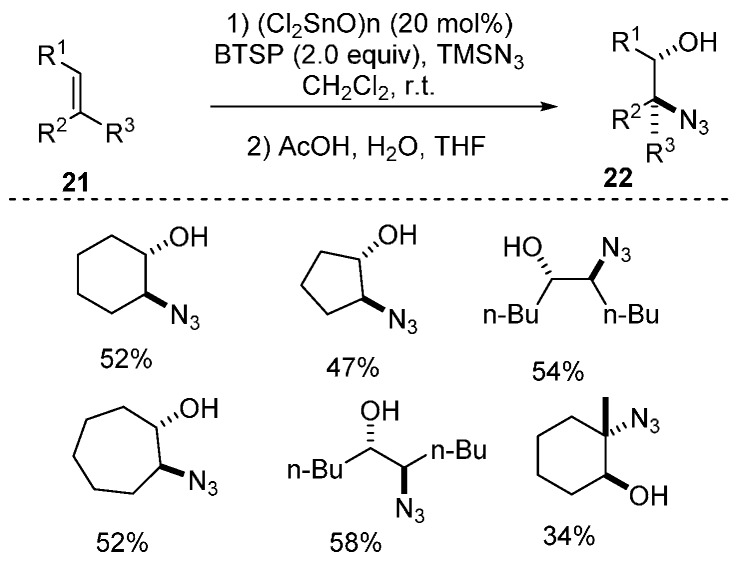
Synthesis of *trans* β-azido alcohols.

Shibasaki and co-workers reported the first example of (Cl_2_SnO)_n_-catalyzed synthesis of *trans* β-azido alcohols by using readily available alkenes as substrates (Scheme 15) [63].

**Scheme 16:**
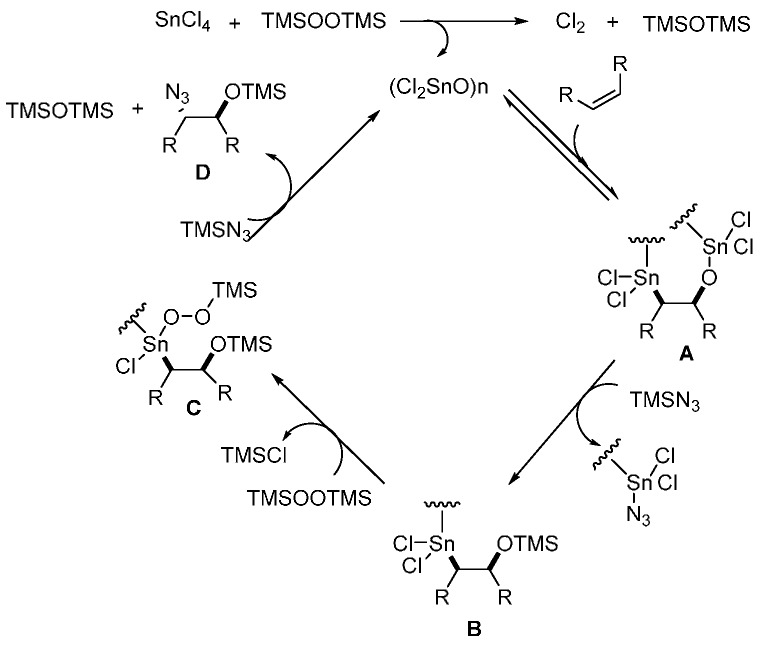
Plausible reaction mechanism.

The authors proposed a catalytic cycle that involves the insertion of a C=C double bond to dichlorotin oxide to afford intermediate **A**, which could react with TMSN_3_ to afford **B**. Subsequent nucleophilic attack of BTSP (bis(trimethylsilyl) peroxide) on Sn gives the intermediate **C.** The regeneration of (Cl_2_SnO)*n* by S_N_2 attack with TMSN_3_ gives **D** as the precursor of the product (Scheme 16).

**Scheme 17:**
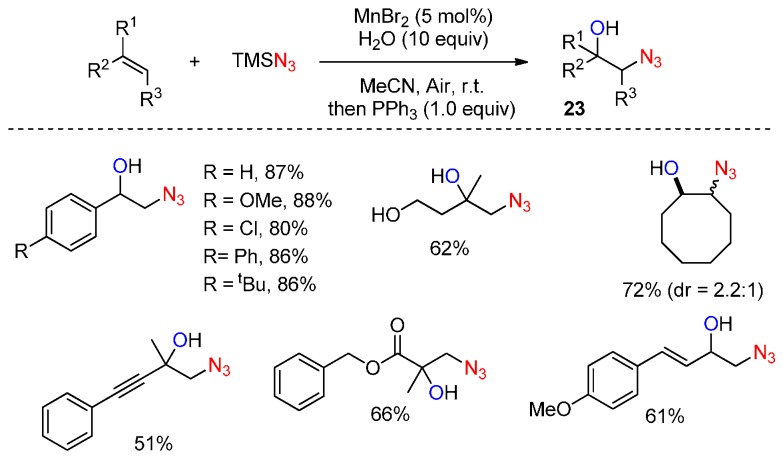
β-Azido alcohol construction.

Recently, the Jiao group reported an efficient Mn-catalyzed aerobic oxidative hydroxyazidation of olefins for the synthesis of β-azido alcohols **23** using air as the oxidant (Scheme 17) [64]. This chemistry was notable for its mild reaction conditions, broad substrate scope, and high reaction efficiency. Moreover, the resulting β-azido alcohols are useful precursors of β-amino alcohols, aziridines, and other O- and N-containing heterocyclic compounds.

**Scheme 18:**
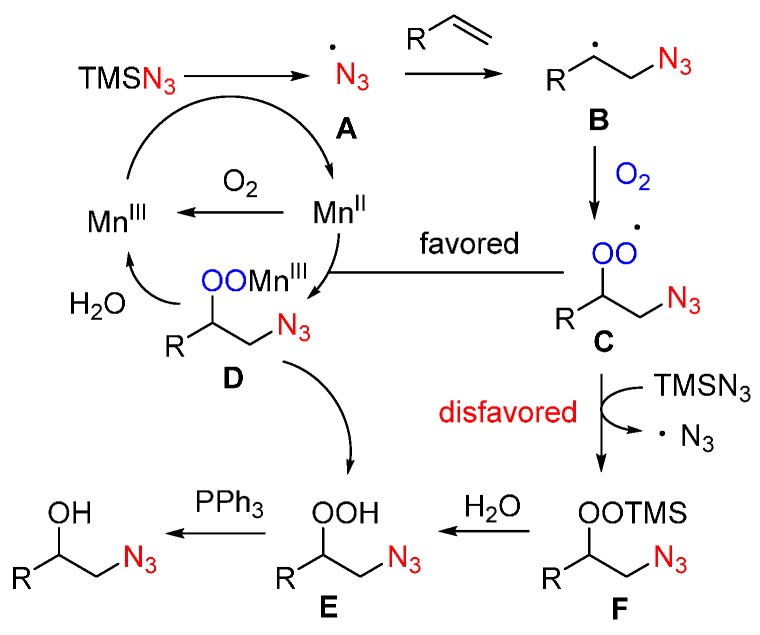
Proposed mechanism.

On the basis of the mechanistic study and density functional theory (DFT) calculations, a favored radical process is proposed (Scheme 18). Firstly, the Mn^II^ is readily oxidized to Mn^III^ or Mn^IV^ by molecule oxygen in the air, then Mn^III^ oxidizes TMSN_3_ to generate azido radical. Mn^IV^ can also oxidize TMSN_3_ to form the N_3_ radical **A** and generate Mn^III^ catalyst. Radical **A** attacks an alkene at the sterically less hindered position to form the carbon radical **B**, which is trapped by oxygen to generate the peroxyl radical **C**. According to the DFT calculations, it is favored for the peroxyl radical **C** to undergo Mn-participated SET and protonation processes to afford β-azido peroxy alcohols **E**. In comparison, the pathway through **F** is disfavored. Finally, β-azido peroxy alcohol **E** is reduced by PPh_3_ to form the β-azido alcohol **23**.

**Scheme 19:**
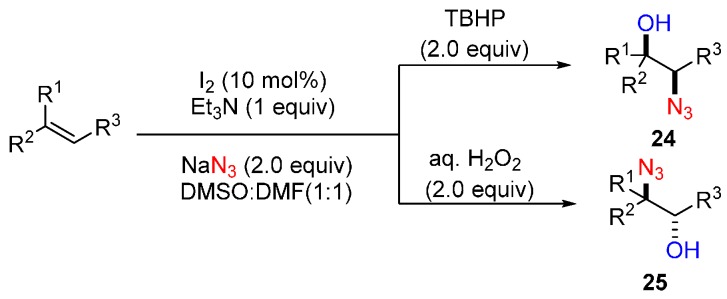
Synthesis of 1,2-azidoalcohols.

Sudalai and co-workers reported an I_2_-catalyzed synthesis of 1,2-azidoalcohols by employing NaN_3_ as N-nuclepohiles and DMF as O-nuclepohiles respectively (Scheme 19) [65]. This approach displayed broad substrate scope and high reaction efficiency with regio- and stereoselectivity. ^18^O labelling studies proved that DMF served as the O-nucleophile. 

**Scheme 20:**
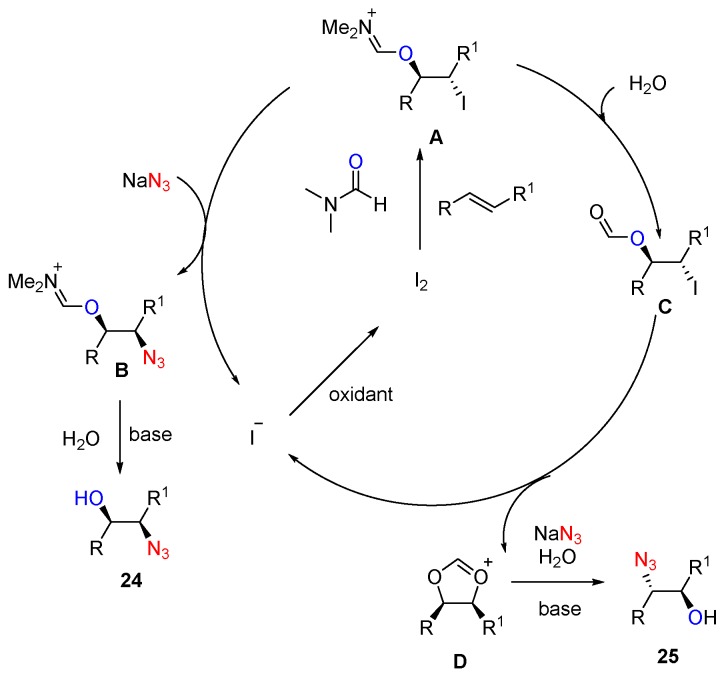
Possible mechanism.

A possible mechanism was proposed (Scheme 20). Firstly, an iodonium ion is formed by the reaction of iodine with alkene, which undergoes subsequent regioselective ring opening with DMF to afford the corresponding iodo intermediate **A**, followed by stereoselective displacement with NaN_3_ to give intermediate **B**. Intermediate **B** on hydrolysis gives *syn* azido alcohols **24**. Alternatively, under aq. H_2_O_2_ conditions, the iodo intermediate **A** is hydrolyzed *in situ* to form iodoformate **C**. The proposed species **D** is formed from **C** by the anchimeric assistance from the formate group, it reacts with the azide anion in a regioselective manner to give *anti* azido alcohols **25** with the liberation of the iodide ion, which is then reoxidized with TBHP/H_2_O_2_ to regenerate I_2_ in the catalytic cycle.

**Scheme 21:**
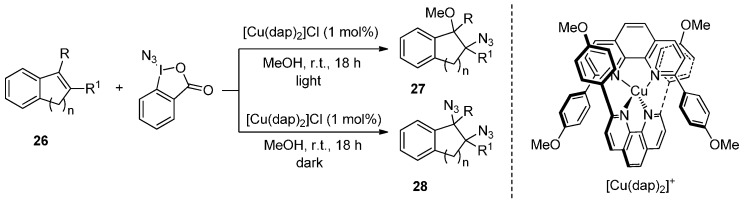
Azidation of styrene-type double bonds.

An effective [Cu(dap)_2_]Cl catalyzed azide addition of styrene-type alkenes **26** using the Zhdankin reagent as N_3_ source was reported by Greaney and co-workers [66]. This is a light-controlled reaction, and in the presence of light, a photoredox cycle is implicated with polar components such as methanol or bromide adding to a benzylic cation. By contrast, in the absence of light, a double azidation takes place, leading to diazide products. Thus, the degree of azidation can be controlled by switching between light and dark conditions (Scheme 21).

**Scheme 22:**
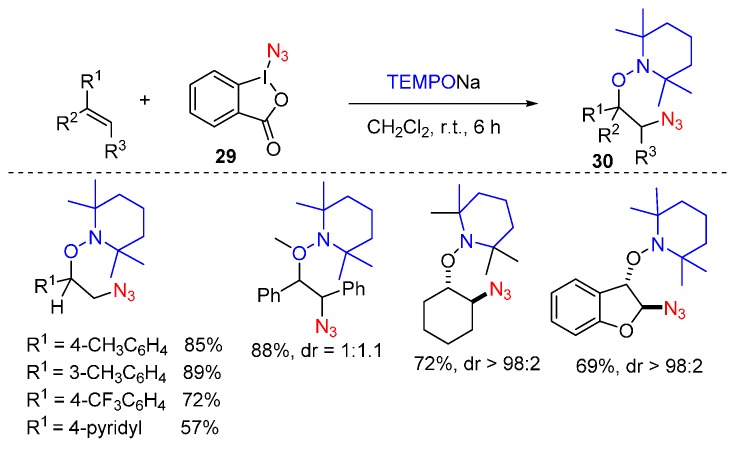
Oxyazidation of alkenes.

Studer and co-worker achieved oxyazidation reactions of alkenes using sodium TEMPO as O source, reagent **29** as N_3_ source under mild condition affording product **30** in good yields (Scheme 22) [67]. It is noteworthy that the oxyazidation of cyclic systems proceeded well with excellent diastereoselectivity.

**Scheme 23:**
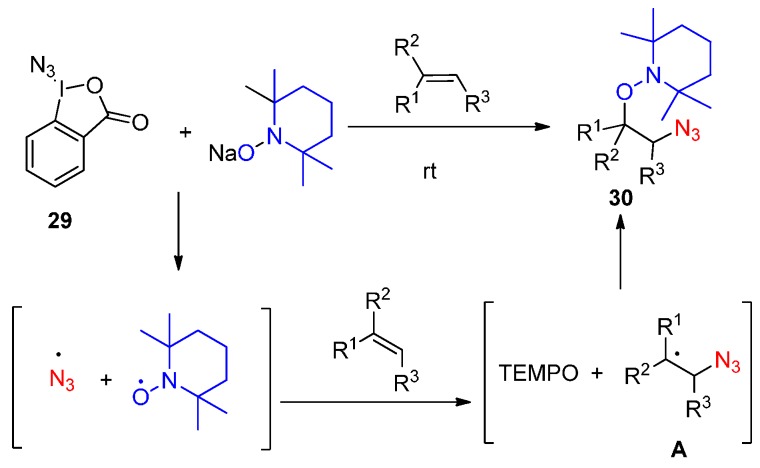
Radical oxyazidation of alkenes.

Inspired by their previous work that TEMPONa could reduce CF_3_-iodine reagent (Togni reagent) to generate CF_3_-radical [68], a radical process was suggested by the authors (Scheme 23). Under the standard reaction conditions, TEMPONa reduced Togni reagent to generate an N_3_ radical, and release TEMPO, then the N_3_ radical is trapped by an olefin to generate species **A**, which reacts with TEMPO to form products **30**.

**Scheme 24:**
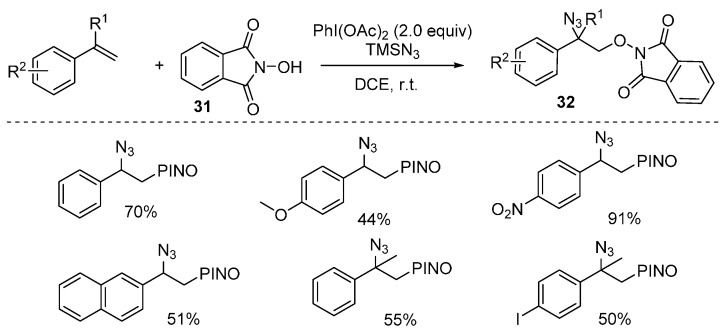
Oxyazidation of alkenes.

Recently, an efficient oxyazidation of alkenes under metal-free conditions was reported by Xia and co-workers. This reaction could form C-O and C-N bonds in one step by using N-hydroxyphthalimide as an oxygen-radical precursor and TMSN_3_ as the N_3_ source. A number of aryl-substituted alkenes was tolerated in this transformation (Scheme 24) [69]. 

**Scheme 25:**
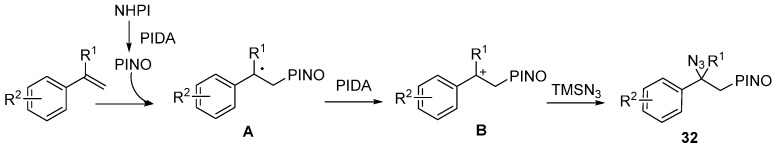
Possible mechanism.

A possible mechanism is outlined in Scheme 25. In the presence of PIDA, N-hydroxyphthalimide (NHPI) **31** could be oxidized to generate an N-oxyl (PINO) radical, which attacks alkenes to form intermediate **A**. Intermediate **A** can be further oxidized to cation intermediate **B** by PIDA. Finally, intermediate **B** is trapped by TMSN_3_ to give products **32.**

**Scheme 26:**
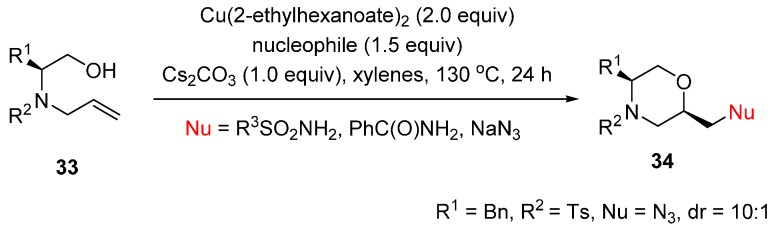
Oxyazidation of alkenes.

The simultaneous addition of oxygen and nitrogen across an alkene is a convenient way to construct the precursor of 2-aminomethylmorpholines **34**. Remarkably, through copper-catalyzed oxyamination of alkene, a novel synthetic method for the preparation of **34** was reported by Chemler and co-workers [70]. Under the reaction conditions, **34** was obtained in good yields with excellent diastereoselectivity (Scheme 26).

**Scheme 27:**
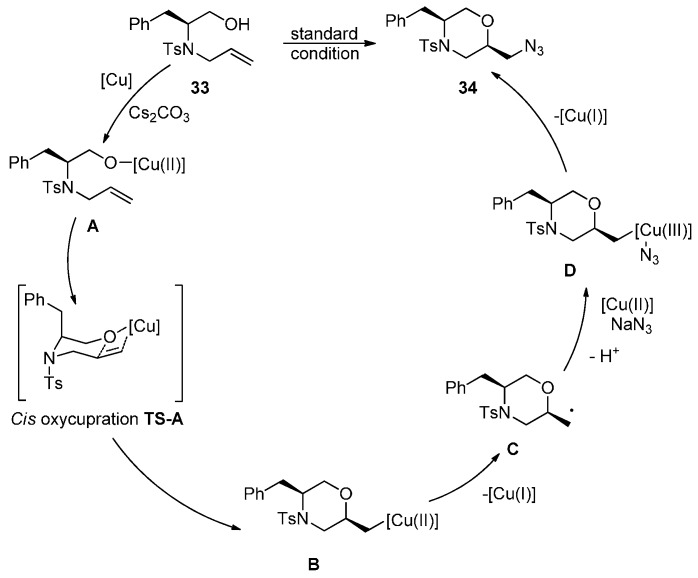
Proposed oxyamination reaction mechanism.

A plausible mechanism is illustrated in Scheme 27. Coordination of alcohol **33** to copper(II) 2-ethylhexanoate gives monomer **A.** Thermal conditions promote *cis-*oxycupration via **TS-A**, then give the copper(II) intermediate **B**. The diastereoselectivity is rationalized by a chair-like transition state where vicinal tosyl and benzyl substituents adopt pseudoaxial positions. Intermediate **B** undergoes C-Cu(II) homolytic cleavage to give carbon radical **C**. In the presence of an azide nucleophile, an organocopper(III) intermediate **D** is formed, which can undergo reductive elimination to form the product **34**.

**Scheme 28:**
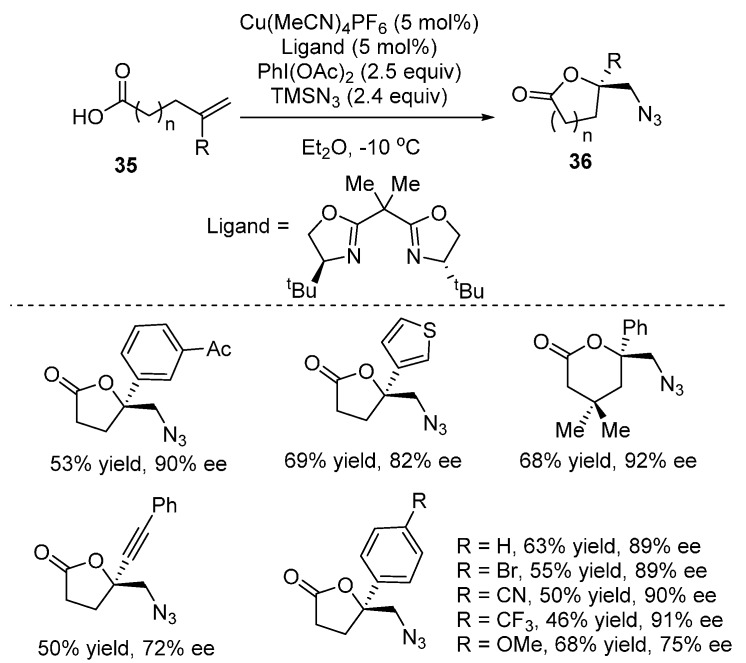
Synthesis of chiral lactones.

A new approach to synthesize a wide range of chiral lactones **36** based on Cu-catalyzed enantioselective radical oxyfunctionalization of alkenes was developed by Buchwald and co-workers [71]. This method provides a straightforward approach to various lactone building blocks containing tetrasubstituted stereogenic centers, which are hard to access through traditional methods (Scheme 28).

**Scheme 29:**
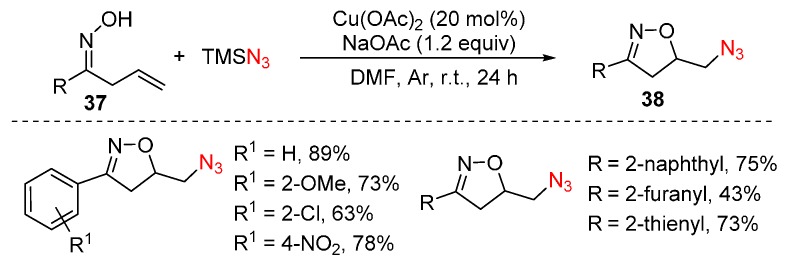
Oxyazidation of alkenes.

Isoxazolines are useful building blocks in organic chemistry [72]. Recently, an efficient Cu(OAc)_2_-catalyzed oxyazidation of alkenes **37** was developed by Wang, Xu and co-workers (Scheme 29) [73]. This reaction occurs under mild conditions, forming the azido-substituted isoxazolines **38** in good yields, although the mechanism of this chemistry is still unclear.

### 2.3. Construction of C-N_3_ Bond and C-N Bond

**Scheme 30:**
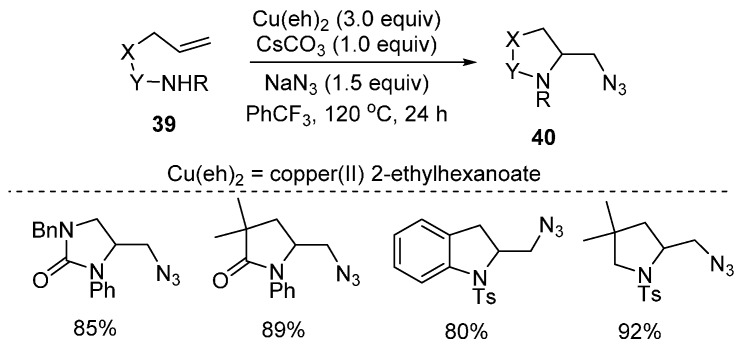
Intermolecular alkene azidoamination.

Olefin diamination methods provide powerful access to vicinal diamines that are useful in chemical biology, medicinal chemistry and materials science. Recently, an novel copper(II)-promoted intramolecular azidoamination of alkenes **39** was reported by Chemler and co-workers (Scheme 30) [74]. This method could tolerate a wide range of internal and external amine sources for the formation of differently functionalized nitrogen heterocycles **40**.

**Scheme 31:**
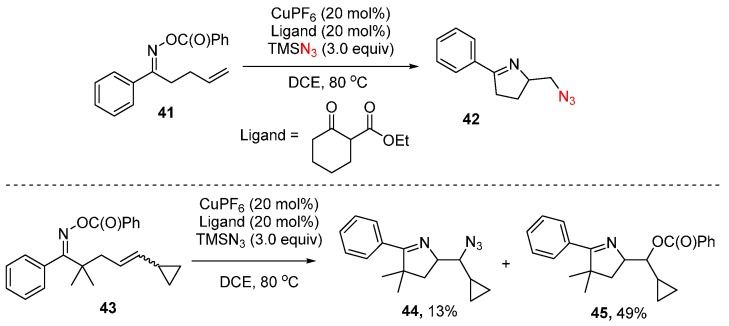
Azidation of unsaturated O-acyl oximes and radical clock experiments.

**Scheme 32:**
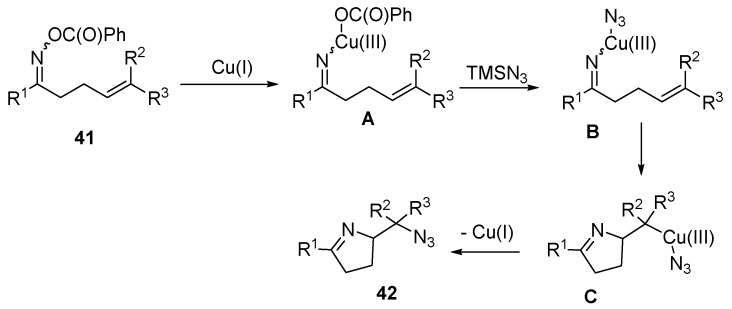
Proposed mechanism.

Yu and co-workers reported an intramolecular azidation reaction through copper-mediated N-O cleavage and subsequent C-N bond forming 5-*exo* cyclization. The forming intermediate is subsequently azidated to afford the corresponding dihydropyrroles (Scheme 31) [75]. To understand this reaction mechanism, compound **43** was treated under standard reaction conditions. However, in this case **44** and **45** are mainly obtained with only trace amounts of ring opening product formation, suggesting that the cyclization step is unlikely a free radical process. Based on these results, a reaction mechanism is proposed (Scheme 32). The first step involves the oxidative addition of Cu(I) to the N-O to give intermediate **A**, which undergoes ligand exchange and then intramolecular cyclization to afford intermediate **C**, followed by reductive elimination to afford products **42**.

**Scheme 33:**
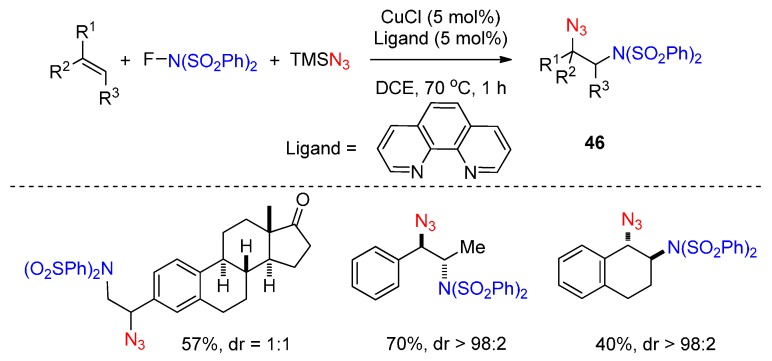
Aminoazidation of alkenes.

Studer and co-workers described a novel methodology for the efficient synthesis of the precursors of vicinal amino azides **46** (Scheme 33) [76], which can easily be transformed into other important amine derivatives. This chemistry employed Cu(I) as catalyst, TMSN_3_ as N_3_ source and available N-fluorobenzenesulfonimide (NFSI) as nitrogen-radical precursor leading to the desired products in moderate to excellent yields with high diastereoselectivity. 

**Scheme 34:**
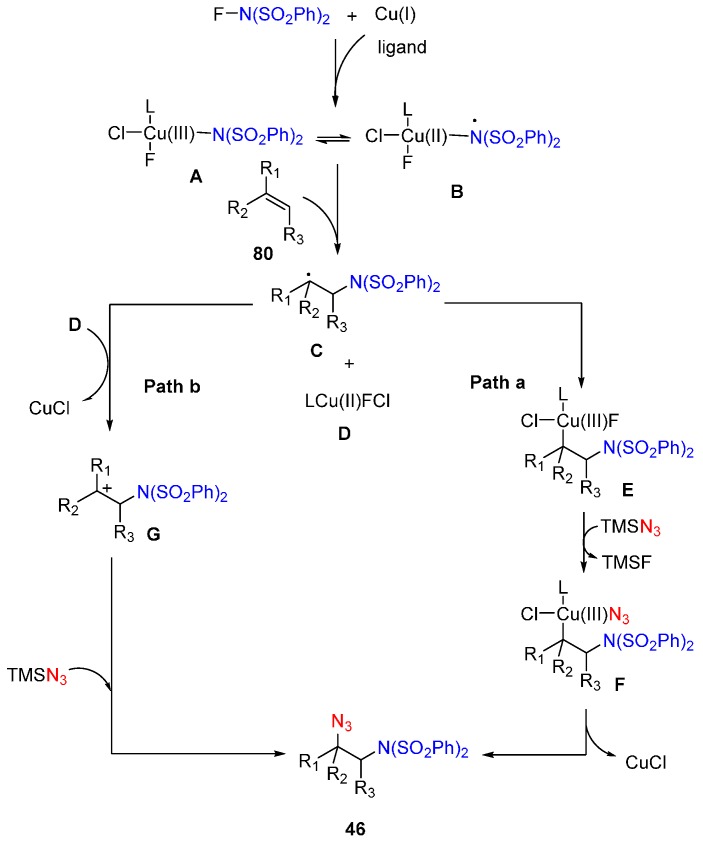
Proposed reaction mechanism.

A plausible reaction mechanism based on the reported processes is outlined in Scheme 34 [77,78]. Firstly, Cu(I) reacts with NFSI to form Cu(III) species **A**, which could exist in equilibrium with a Cu(II)-stabilized N-centered radical **B**. It is the precursor of bis-sulfonylamidyl radical, which adds to the alkene to generate carbon radical **C** and Cu(II) species **D**. Also **B** species could react as N-radicals with the alkene. Then two pathways are suggested. In path a, trapping of **C** with **D** provides Cu(III) species **E**, which exchanges ligand with TMSN_3_ to give Cu(III) complex **F**. Reductive elimination of **F** affords the products along with the regeneration of the Cu(I) catalyst. In path b, **D** oxidizes **C** to cationic intermediate **G**, which is trapped by TMSN_3_ to form the products. 

**Scheme 35:**
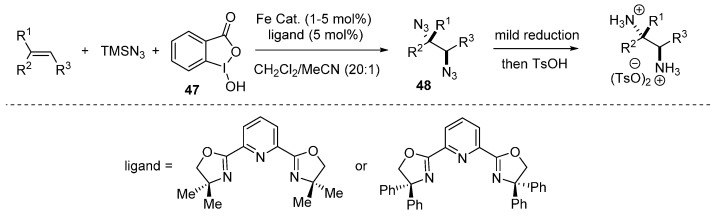
Diazidation of olefins.

Snider and co-workers reported a Mn(OAc)_3_-mediated diazidation of alkenes and glycals for the formation of 1,2-diazides compounds [79]. Very recently, Xu and co-workers reported a novel iron-catalyzed diastereoselective olefin diazidation method which tolerates a broad range of olefins (Scheme 35) [80]. This method also provides a convenient approach to vicinal primary diamines and other synthetically valuable nitrogen-containing compounds.

**Scheme 36:**
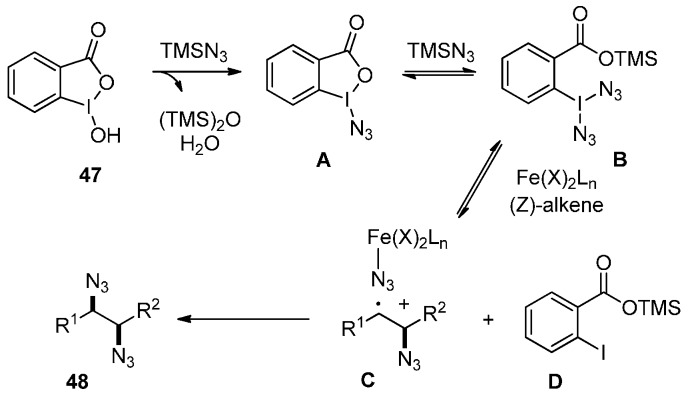
Possible mechanism.

A proposed mechanism is shown in Scheme 36. TMSN_3_ reacts with **47** to give intermediate **A**, which is further activated by TMSN_3_ to generate intermediate **B**. In the presence of iron catalysts, intermediate **B** reacts with alkene to afford intermediate **C** and release intermediate **D**. It is likely that the high-valent iron species may be further oxidize intermediate **C** through inner-sphere azido ligand transfer to afford the diazide product.

### 2.4. Construction of C-N_3_ Bond and C-P Bond

**Scheme 37:**
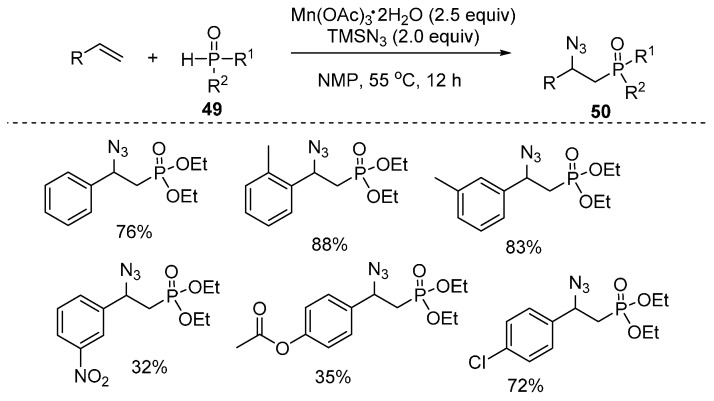
Phosphonation–azidation of alkenes.

An efficient method for the synthesis of β-azidophosphonates **49** through Mn(OAc)_3_-mediated radical oxidative phosphonation-azidation of alkenes under relatively mild reaction conditions was reported by Tang and co-workers. This reaction displayed a broad substrate scope and can be easily scaled up. The products can be obtained in a one-pot operation (Scheme 37) [81].

**Scheme 38:**
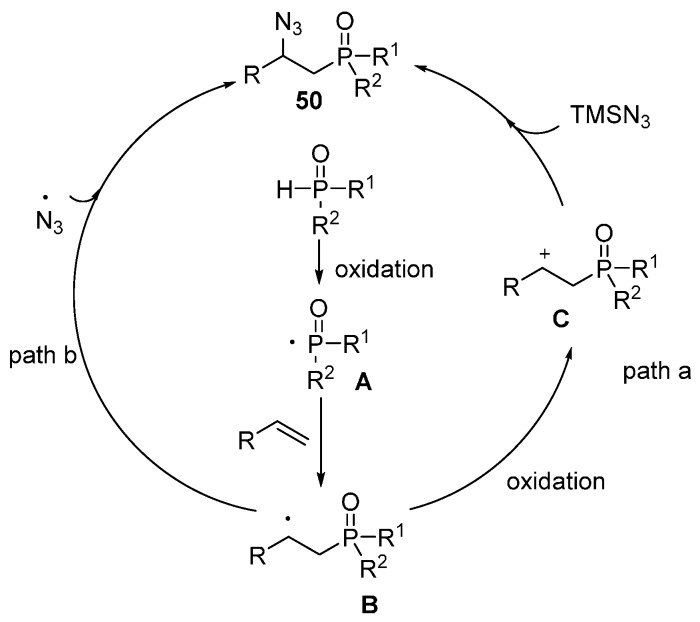
Possible reaction pathways.

Based on their previous studies in P–C bond formation, and reaction of organophosphorus radicals [82], a possible mechanism was proposed (Scheme 38). The reaction is initiated by the addition of phosphorous radical **A** to alkene to generate radical **B**, which undergoes further oxidation to afford cationic intermediate **C**. The intermediate **C** is then trapped by TMSN_3_ to afford the final product (path a). Alternatively, the radical **B** could be directly trapped by an azido radical to form the product (path b) (Scheme 38).

### 2.5. Construction of C-N_3_ Bond and C-Se Bond

**Scheme 39:**
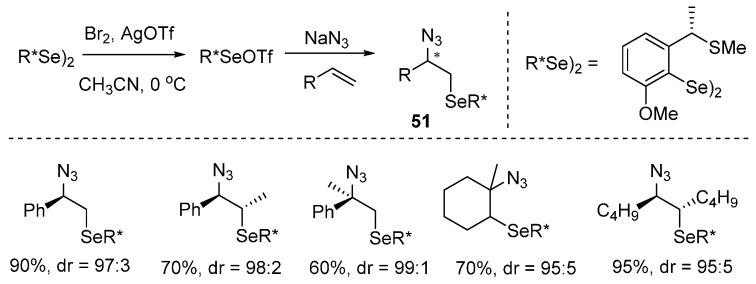
Azidoselenenylation of alkenes.

Tiecco and co-workers reported the first example of a highly asymmetric electrophilic azidoselenenylation of olefins for the preparation of azido selenium derivatives **51**. This reaction occurs with a high level of facial selectivity, which was made possible by the use of chiral, non-racemic selenium reagents (Scheme 39) [83].

### 2.6. Construction of C-N_3_ Bond and C-Halogen Bond

**Scheme 40:**
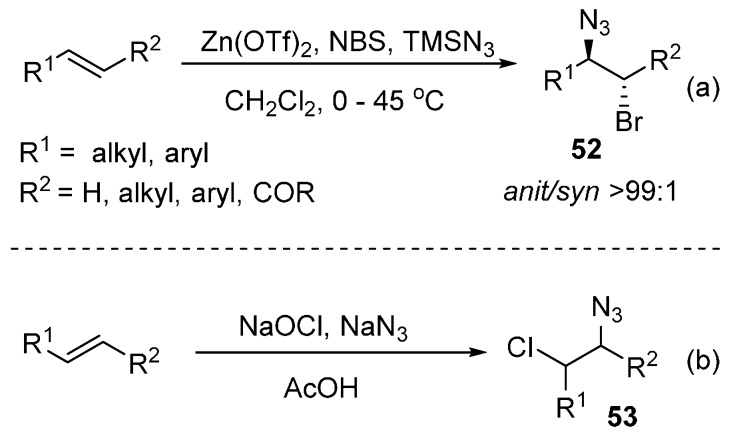
Bromoazidation or chloroazidation of alkenes.

1,2-Haloazidation of alkenes represents an important transformation in organic synthesis. By using Zn(OTf)_2_ as catalyst, a metal-catalyzed bromoazidation of alkenes was reported by Hajra and co-workers. The corresponding bromoazidation products were obtained in good yields by using this protocol (Scheme 40a) [84]. Phukan’s group also achieved the bromoazidation of alkenes by using other bromine sources [85,86]. Moreover, a route to 1,2-azidochlorides from alkenes was developed by Finn and co-workers (Scheme 40b) [87].

## 3. Conclusions

In conclusion, this review has summarized recent difunctionalization reactions of olefins with organic and inorganic azides through C-N_3_ bond formation. An oxidative single electron transfer (SET) process is involved in most cases. These approaches provide efficient protocols for the preparation of various organic azido compounds, which can then be further applied in many transformations to synthesize various valuable nitrogen-containing compounds.
